# Treatment of Goitre with Ergotin

**Published:** 1882-05

**Authors:** 


### Treatment of Goitre with Ergotin.

M. Bauwens, speaking of the treatment of goitre with ergotin, divides cases of goitre into the following classes: 1. Cystic goitre, with easily apparent fluctuation. 2. Goitre partly cystic and partly hypertrophic. 3. Goitre characterized by diffuse parenchymatous hypertrophy and great vascularity. 4. Recent goitre, soft and diffuse. In cystic goitre, and in soft, diffuse, recent goitre, the author considers that iodine is the best remedy, and has most confidence in parenchymatous injection as the mode of its employment. He calls attention to the fact, however, that in proportion as vascularity predominates as a cause of thyroid enlargement, so will iodine fail to cause reduction. Iodine stimulates the reabsorption of the contents of the cysts, but can not cause the vascularity to diminish. It is in these latter cases that the author recommends ergotin. The ergotin should be injected into the substance of the tumor; it at once causes contraction of the muscular coats of the small arteries, and a diminution in the size of the tumor and in the amount of pulsation observed in it is at once apparent. He uses the following solution: ℞. Yvon's ergotin, 1 gramme (gr. xv); glycerine, water, in equal parts, enough to make 7 grammes (ʒ j¾). Of this solution he injects 2 grammes (ʒ ss.) at a time directly into the substance of the goitre; this treatment is repeated at intervals of about two weeks until a cure is effected. It may not be out of place to mention that the ergotin of Yvon does not differ in any essential from that of Bonjean and other makers. The author reports the following case: A woman of twenty-nine had suffered from goitre since the age of fourteen. Moderate in size for seven or eight years, the tumor had lately begun to enlarge quite rapidly; at the time of examination it measured three inches transversely, by two and a half vertically, the right lobe predominating. It was elastic to the touch, pulsated, and presented a slightly marked souffle. At the menstrual epoch, upon excitement, or as a result of singing, shouting, or hard work, the tumor became more enlarged, and pulsated vigorously. The signs were those of a goitre essentially hypertrophic, with predominance of the vascular element. At times there were attacks characterized by dyspnœa, buzzing in the ears, dizziness, and dimness of vision. Twice the patient had suffered from attacks of complete aphonia lasting two or three weeks. Iodine had been tried in various forms and ways, but without any good result. Parenchymatous injections of ergotin, as described above, were practiced for five weeks every six or seven days; at the end of that time the tumor had completely disappeared. There was at no time any considerable soreness or pain as a result of the injections. A little swelling with some sensitiveness at the point of injection, lasting only about forty-eight hours, was the only trouble occasioned. The author suggests the treatment in exophthalmic goitre, although he has not yet had an opportunity to try it.—*N. Y. Med. Journ. and Obst. Rev.*

